# A Pilot Study: The UNC Passive Aerosol Sampler in a Working Environment

**DOI:** 10.1093/annweh/wxx067

**Published:** 2017-08-05

**Authors:** Mariam Shirdel, Håkan Wingfors, Britt M Andersson, Johan N Sommar, Ingvar A Bergdahl, Ingrid E Liljelind

**Affiliations:** 1 Occupational and Environmental Medicine, Department of Public Health and Clinical Medicine, Umeå University, 901 87, Umeå, Sweden;; 2 Swedish Defence Research Agency, CBRN Defence and Security Division, Cementvägen 20, SE-901 82, Umeå, Sweden;; 3 Department of Applied Physics and Electronics, Umeå University, SE-901 87, Umeå, Sweden

**Keywords:** inorganic dust, PM_2.5_, PM_10_, scanning electron microscopy, UNC passive aerosol sampler, working environment

## Abstract

**Objectives:**

Dust is generally sampled on a filter using air pumps, but passive sampling could be a cost-effective alternative. One promising passive sampler is the University of North Carolina passive aerosol sampler (UNC sampler). The aim of this study is to characterize and compare the UNC sampler’s performance with PM_10_ and PM_2.5_ impactors in a working environment.

**Methods:**

Area sampling was carried out at different mining locations using UNC samplers in parallel with PM_2.5_ and PM_10_ impactors. Two different collection surfaces, polycarbonate (PC) and carbon tabs (CT), were employed for the UNC sampling. Sampling was carried out for 4–25 hours.

**Results:**

The UNC samplers underestimated the concentrations compared to PM_10_ and PM_2.5_ impactor data. At the location with the highest aerosol concentration, the time-averaged mean of PC showed 24% and CT 35% of the impactor result for PM_2.5_. For PM_10_, it was 39% with PC and 58% with CT. Sample blank values differed between PC and CT. For PM_2.5_, PC blank values were ~7 times higher than those of CT, but only 1.8 times higher for PM_10_. The blank variations were larger for PC than for CT.

**Conclusions:**

Particle mass concentrations appear to be underestimated by the UNC sampler compared to impactors, more so for PM_2.5_ than for PM_10_. CT may be preferred as a collection surface because the blank values were lower and less variable than for PC. Future validations in the working environment should include respirable dust sampling.

## Introduction

Passive sampling for gaseous compounds, using tube and badge-type samplers, has long been an established technique in occupational exposure assessment studies (Nieuwenhuijsen, 2003). In contrast, passive sampling of aerosols in working environments has not been studied as extensively, for example Vinzents (1996), Wagner and Leith (2001), and Vinzents *et al.* (2001) applied passive sampling in working environments. The active sampling with gravimetric determination commonly used is expensive because of labour costs, and requires the expertise of occupational hygienists. Passive sampling minimizes time and cost and could be an alternative for occupational hygienists. The University of North Carolina (UNC) sampler is a passive aerosol sampler that has been used in ambient environments for characterisation of particulate matter with aerodynamic particle size below 10 µm (PM_10_) and 2.5 µm (PM_2.5_) (Wagner and Leith, 2001, 2001a, 2001b; Wagner and Macher, 2003; Leith *et al.*, 2007; Ott and Peters, 2008; Whitehead and Leith, 2008). Measurements have often been carried out for weeks, ranging from 3 hours to 5 weeks. In contrast, the sampling time in occupational environments are often limited to 8 hours.

The aim of this study was to make a first characterisation of the UNC sampler’s stationary performance in an occupational mining environment. We tried different collection surfaces, sampler exposure times, and compared the UNC sampler’s results to impactors for PM_10_ and PM_2.5_ in a wide concentration range and sampling for 4 to 25 hours.

## Material and Methods

**Figure 1.**
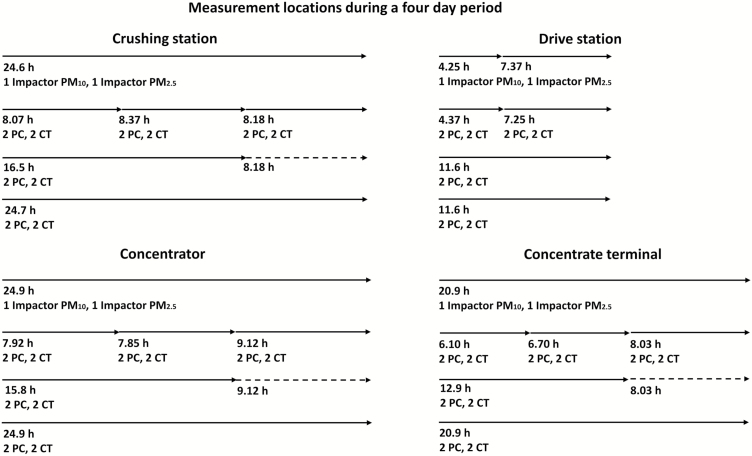
Overview of the sampling schedule at the four locations during a 4-day period for the impactors: PM_10_ and PM_2.5_; and UNC samplers: polycarbonate- (PC) or carbon tab (CT) collection surface. An arrow represents a sampling occasion. A dashed arrow represents re-using a sampling occasion to enable comparison between measurements for the same time period.

Area sampling was made at four locations in an open-pit mine: a crushing station, a drive station, a concentrator, and a concentrate terminal. With the exception of the drive station, a 24-hour period was covered at each location (Fig. 1). Time-averaged means of the concentrations from the samplers (and at one location impactors as well) were calculated to be able to compare the whole measurement durations (ca. 24 hours, except for drive station: ca. 12 hours). The crushing station was located outdoors 165 metres below ground level, while the other three were indoors. Due to high dust emissions, the floor was watered at the concentrator. We used UNC samplers with either the polycarbonate (PC) or carbon tab (CT) collection surface in parallel with impactors measuring PM_10_ and PM_2.5_ (Fig. 1). In total, 10 PC and 10 CT UNC samplers were used at each location. Single PM_10_ and PM_2.5_ impactor collections were made. At the drive station, sampling was carried out over a shorter time due to expected high exposure and risk of overload; thus, the filters were changed once for the impactors.

The wind speed and humidity were measured with a weather station containing a hygrometer and a cup anemometer at 30-minute intervals. The temperature was measured with one ACR SmartButton at 5-minute intervals.

### Active sampling

Impactors (SKC Inc., Eighty Four, PA, USA) with a diaphragm pump (Gast manufacturing, Inc., MI, USA) delivering flows at 10 ± 0.5 litre min^−1^ by the use of adjustable restrictors and PTFE membrane filters (Zeflour, 47 mm, 2.0 µm, Pall, USA) were used for the sampling of PM_10_ and PM_2.5_. A primary flow meter (DC-Lite, Bios International, NJ, USA) was used to measure all air flows at the beginning and end of each sampling period. A Lighthouse HH 3016-IAQ particle counter measuring particles with an aerodynamic diameter of 0.3–10 µm was also used 15 min at the start of each sampling and 15 min at the end. The result from the Lighthouse was converted to PM_0.5_, PM_1.0_, PM_2.5_, PM_5.0_, and PM_10_ for the density of 2.0 g cm^−3^. PM_0.75_ was derived from the mean of PM_0.5_ and PM_1.0_.

Before and after the sampling, the filters and the 14 transport blanks were weighed twice in a laboratory at room temperature. The filters were stored and transported in sealed protective holders.

### Passive sampling

The UNC sampler (Wagner and Leith, 2001a) consists of a holder, a 12 mm aluminium scanning electron microscopy (SEM) stub (Ted Pella Inc., Redding, CA, USA) with a collection surface under a mesh cap with 150 µm (top) and 228 µm (bottom) conical holes. The PC UNC sampler’s (RJ LeeGroup, Monroeville, PA, USA) collection surface consists of a layer of Ted Pella Electrodag DAG-T-502 Carbon paint (Ted Pella Inc.) on the stub and covered with 1/20 of a polycarbonate filter 47 mm Millipore 0.1 µm pore (Merck Millipore, Darmstadt, Germany). The CT UNC sampler’s collection surface was mounted in-house by applying a 12 mm ‘leit adhesive carbon tab’ (Agar Scientific, Essex, UK). This was done in a cleanroom ISO class 6.

Following Ott and Peters (2008), the UNC samplers were protected by a flat plate (provided in kind by RJ Lee Group) placed 1.58 cm above a mounting plate for the samplers, at all locations. We modified the construction in order to ensure electrical connection between the stubs and the mounting plate: A wire connected the bottom plate to ground, and electrically conductive copper tape was applied inside the mounting holes for the stubs.

#### Microscopy, image processing, and conversion to concentration

The analysis method and equations used for the UNC sampler have previously been described by Wagner and Leith (2001a), Schneider *et al.* (2002), Leith *et al.* (2007), and Ott and Peters (2008). The collection efficiency curves from Hinds (1999) were used for each particle fraction, as previously described by Ott and Peters (2008). The UNC samplers were analysed with a Philips XL30 ESEM Scanning Electron Microscope D1079 with a solid state backscatter detector, and the settings were: Beam voltage: 20 kV; spot size: 5.0; magnification: 100×; working distance: 10.8 mm; and vacuum Aux: 0.7 torr. The whole area of the UNC samplers was captured, requiring 24–44 images for PC and 16–37 images for CT, with typical images shown in Supplementary Fig. S1 (available at *Annals of Work Exposures and Health* online).

The images were processed with ImageJ (Version 1.48, National Institutes of Health, USA, released 2014) and MATLAB [R2014b (8.4.0.150421), The MathWorks, Inc., Natick, MA, USA, released 2014]. The threshold method was set to RenyiEntropy; minimum area: 0.41 µm^2^; and maximum area: 10000 µm^2^. The volume shape factor, dynamic shape factor, and density were set to 1.6, 1.4, and 2.0 g cm^−3^, respectively (Wagner and Leith, 2001a; Wagner and Macher, 2003).

#### Blanks

During mounting and dismantling, one PC and one CT field blank at each location were opened at each start of sampling for ~5 min each, to account for contamination from manual handling. There were also two transport blanks (never opened) for each type of UNC sampler. The field blank mean was calculated separately for the PC and CT samplers and subtracted from all results. When the subtraction resulted in negative values, these were not excluded or corrected, as that would bias mean values and blank variation estimates.

## Results and Discussion

### Blanks

**Figure 2.**
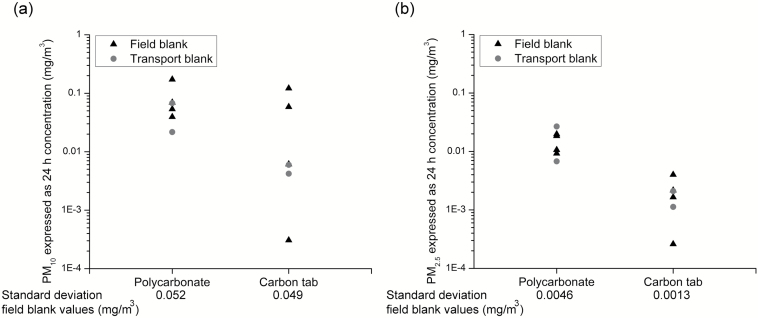
Field and transport blank values expressed as 24-hour concentrations in mg m^−3^ for polycarbonate- and carbon tab collection surfaces. The standard deviation for the field blanks are also noted. (**a**) PM_10_. (**b**) PM_2.5_.

**Figure 3.**
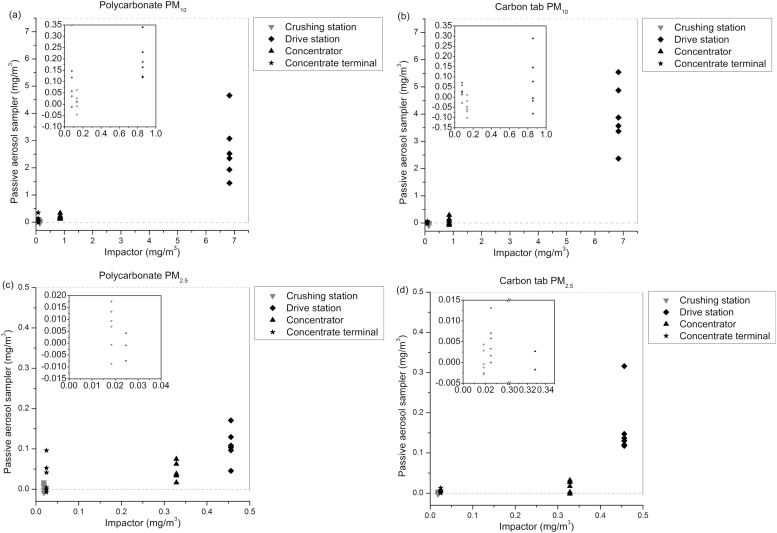
Time-averaged UNC sampler versus impactor concentrations. Figure insertions show a magnification for low concentrations: (**a**) polycarbonate PM_10_; (**b**) carbon tab PM_10_; (**c**) polycarbonate PM_2.5_; and (**d**) carbon tab PM_2.5_.

There was a good agreement between field and transport blank values (Fig. 2). The PC UNC sampler blank values for PM_2.5_ were ~7 times higher than those of CT UNC sampler blanks, while the difference for PM_10_ blanks was smaller at 1.8. In parallel, the standard deviation for the PM_2.5_ blanks was about three times higher for PC than CT UNC samplers, but there was no significant difference for PM_10_ blanks. Apparently, the PC samplers were more contaminated before sampling than the CT samplers. Blank subtraction is not always done with the UNC sampler (Sawvel, 2013). The blank subtractions explain the negative values in Fig. 3.

### Comparison with impactor

Compared to PM_10_ and PM_2.5_ impactor data, the UNC samplers underestimated the concentrations (Fig. 3). The underestimation was most striking for PM_2.5_: the time-averaged mean of PC showed only 24% of the impactor result at the drive station, which was the location with the highest concentration. The corresponding result for CT was 35%. The underestimation was even larger at the concentrator. However, for PM_10_ it was more moderate, with PC showing 39% and CT 58% of the impactor results. The choice of using blank subtraction did not on the whole affect the underestimation. The numbers above changed to 34, 37, 43, and 60%, respectively, when not subtracting blank values.

The PC PM_2.5_ data showed a larger variation than the CT data. This was most visible at low concentrations (Fig. 3c and d), suggesting that contamination already before sampling could be a reason. This suspicion is supported by the observation of higher, as well as variation in, blank values for PM_2.5_ on PC-surface compared to CT (Fig. 2b, standard deviation for PC- 0.0046 mg m^−3^ versus CT blank values 0.0013 mg m^−3^).

### Limitations

Previous studies in the ambient environment have shown lower particle concentrations for the UNC passive sampler compared to impactors and federal reference methods (FRM), especially for the smaller particles, but not that consistent for larger particles (Wagner and Macher, 2003; Watkins *et al.*, 2009; Arashiro and Leith, 2013). The reasons appear to be unknown. Possible mechanisms involved may be wind turbulence, electrostatic forces, condensed water droplets washing away particles, loss from sampler (at sampling or transport), turbulence in the SEM chamber, evaporation due to the vacuum in the SEM chamber, and imaging (resolution).

With regard to wind and turbulence, wind speed was registered at 0 m s^−1^ in all locations. Therefore, this should not be the cause here. Electrostatic forces may repel particles from the sampler if the sampler and particles are charged in the same way. We grounded the samplers, minimising the risk of electrostatic effects. Water droplets may explain the large underestimation at the concentrator, where watering was used for dust control. This gave rise to traces of dried water visible on the CT collection surface with a geometry that mirrored that of the mesh cap; the water may have washed away particles. Regarding loss of particles in transport, the samplers were kept in a horizontal position during transport. They were placed in a soft foam (mattress) with holes for the holders in order to minimize vibrations. Evaporation of substance due to the vacuum in the SEM chamber appears impossible for the mineral particles originating from a mine. We cannot exclude that small particles are lost in turbulence at evacuation. If that occurs, then PC should be more affected than CT, as the latter is stickier.

Counting statistics was not a problem. All samplers had at least 55 counts for PM_2.5_ and 71 counts for PM_10_. We therefore consider counting statistics’ contributions as negligible. Insufficient image resolution of particles smaller than or with the area of one pixel could lead to an underestimation, in this study the pixel side length was 0.64 µm. The choice of shape factors might also affect the underestimation. In this pilot study, the intention was to use the passive sampler in a working environment. An occupational hygienist would not have prior knowledge of the particles nor the time to conduct analysis at different magnifications for several samplers. Thus, for this study, with particles of different size and shapes, the model with shape factors for heterogeneous aerosols from Wagner and Macher (2003) seemed most appropriate and was therefore applied. It should be noted that the recommended image analysis method for particles between 0.1 and 10 µm by Wagner and Macher (2003) was not followed.

The possibility of underestimation because of image resolution was investigated by using the result from the Lighthouse. We regarded particles smaller than 0.75 µm as potentially undetected in the microscope and therefore estimated the percentage of such particles in PM_2.5_ and PM_10_. The ratios PM_0.75_/PM_2.5_ and PM_0.75_/PM_10_ showed that the magnification may be somewhat contributing (Table 1), but not to an extent that could explain the degree of the underestimation for both PM_2.5_ and PM_10_.

**Table 1.** PM ratios for PM_0.75_/PM_2.5_ and PM_0.75_/PM_10_ from the Lighthouse for each location.

Location	PM_0.75_/PM_2.5_	PM_0.75_/PM_10_
Crushing station	16%	1.2%
Drive station	4.1%	0.073%
Concentrator	14%	3.6%
Concentrate terminal	23%	2.1%

## Conclusions and Implications for Future Studies

The UNC sampling analysis method appears to underestimate PM_2.5_ concentrations compared to PM_2.5_ impactors at relatively low microscope resolutions. For PM_10_, we also observed lower values compared to impactor sampling, but the underestimation was more moderate and previous studies vary in this sense. For the potential in occupational environments, the underestimation of PM_2.5_ gives rise to concern. Furthermore, although the UNC sampler may be easy to use and cost-effective for collection, the subsequent analysis with a non-automated SEM is time consuming and costly. There are, however, advantages with passive samplers. It would, therefore, be of interest to characterize the UNC sampler’s performance in relation to respirable dust samplers, as these are generally used in working environments. In such future studies, we may prefer CT as a collection surface because the blank values were lower and less variable than those of the PC surfaces we used here. They are also easier to assemble. We also suggest carrying out repeated measurements with the impactors. We used only one impactor for PM_10_ and one for PM_2.5_, not giving any information of the variation, which may be significant. In order to gain more information on how underestimation is related to particle size distribution, this can be studied in parallel to UNC sampling. A higher magnification for the microscope imaging can also be applied. Lastly, one should avoid locations with very high humidity, as water condensation and precipitation may affect the UNC sampler.

## Supplementary Data

Supplementary data are available at *Annals of Work Exposures and Health* online.

## Supplementary Material

Supplementary Material
