# Supplementary material for: A Pilot Study: The UNC Passive Aerosol Sampler in a Working Environment
Source: Ann Work Expo Health. 2017 Aug 5;61(8):1029–34. doi: 10.1093/annweh/wxx067 (PMC6824523; doi:10.1093/annweh/wxx067)
Supplement: Supplementary Material [file wxx067_suppl_supplementary_material.pdf]

A pilot study: The UNC passive aerosol sampler in a working environment

M. Shirdel[1], H. Wingfors[2], B. M. Andersson[3], J. N. Sommar[1], I. A. Bergdahl[1], and I. E. Liljelind[1]

[1]Occupational and Environmental Medicine, Department of Public Health and Clinical Medicine, Umeå University, 901 87, Umeå, Sweden
[2] Swedish Defence Research Agency CBRN Defence & Security Division, 901 82, Umeå, Sweden
[3] Department of Applied Physics and Electronics, Umeå University, 901 87, Umeå, Sweden

SUPPLEMENTARY MATERIAL

Figure S1 shows typical scanning electron microscopy images of the particles collected with the UNC sampler at the different measurement locations in the open-pit mine.

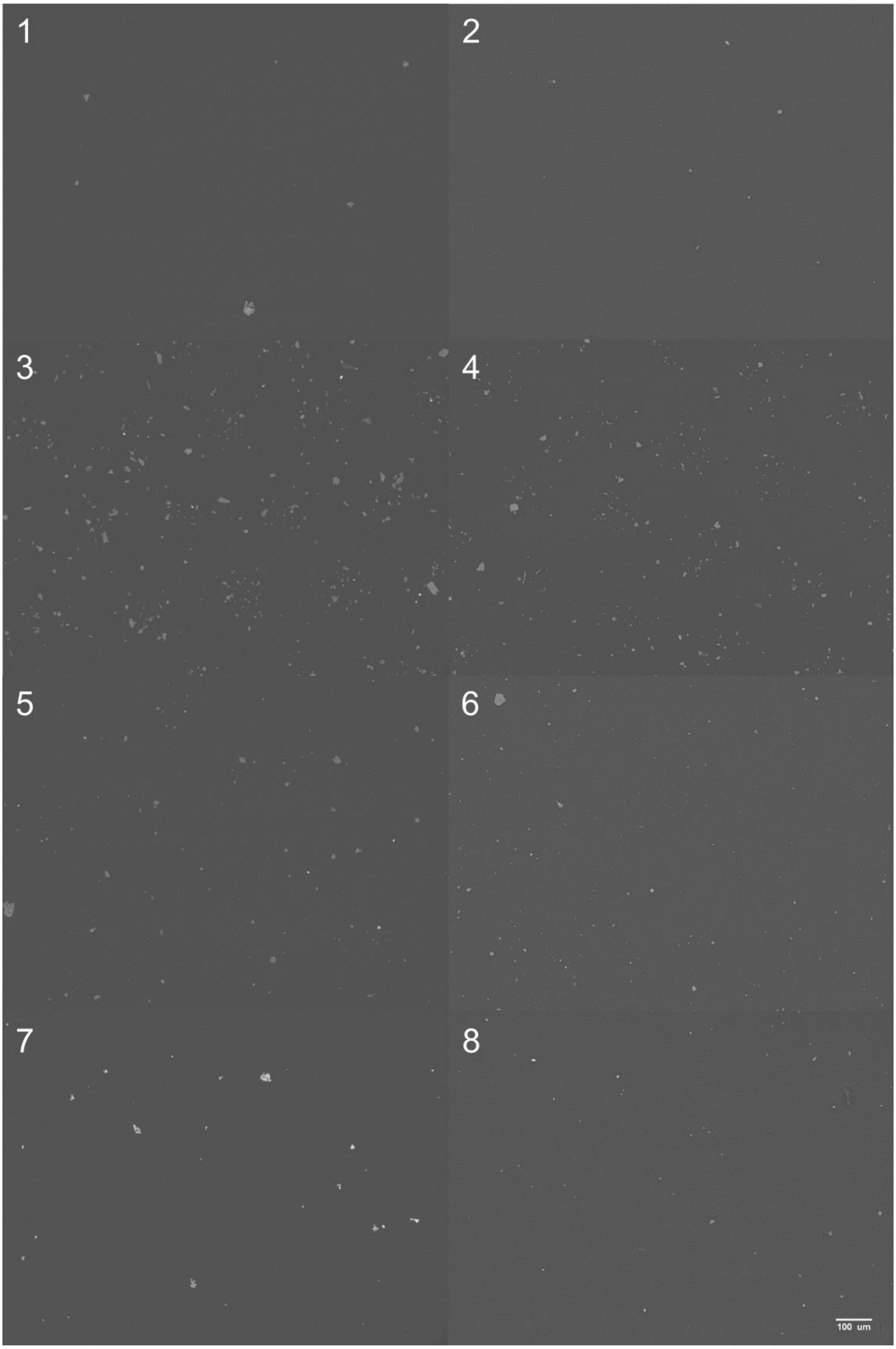

Figure S1. An example of scanning electron microscopy images of the particles collected by the UNC sampler at the different measurement locations in the open-pit mine; **1.** Crushing station UNC sampler with carbon tab; **2.** Crushing station UNC sampler with polycarbonate; **3.** Drive station UNC sampler with carbon tab; **4.** Drive station UNC sampler with

polycarbonate; **5.** Concentrator UNC sampler with carbon tab; **6.** Concentrator UNC sampler with polycarbonate; **7.** Concentrate terminal UNC sampler with carbon tab; **8.** Concentrate terminal UNC sampler with polycarbonate.
